# Inhibition of Matrix Metalloproteinase 9 Enhances Rod Survival in the S334ter-line3 Retinitis Pigmentosa Model

**DOI:** 10.1371/journal.pone.0167102

**Published:** 2016-11-28

**Authors:** Jung-A Shin, Hwa Sun Kim, Andrew Vargas, Wan-Qing Yu, Yun Sung Eom, Cheryl Mae Craft, Eun-Jin Lee

**Affiliations:** 1 Mary D. Allen Laboratory for Vision Research, USC Roski Eye Institute, Department of Ophthalmology, Keck School of Medicine of the University of Southern California, Los Angeles, California, United States of America; 2 Department of Anatomy, School of Medicine, Ewha Womans University, Seoul, Republic of Korea; 3 Department of Cell & Neurobiology, Keck School of Medicine of the University of Southern California, Los Angeles, California, United States of America; 4 Department of Biological Structure, University of Washington, Seattle, Washington, United States of America; 5 Neuroscience Graduate Program, University of Southern California, Los Angeles, California, United States of America; 6 Department of Biomedical Engineering, University of Southern California Viterbi School of Engineering, Los Angeles, California, United States of America; University of Tennessee Health Science Center, UNITED STATES

## Abstract

Retinitis Pigmentosa (RP) is one of the most common forms of inherited visual loss with the initial degeneration of rod photoreceptors, followed by a progressive cone photoreceptor deterioration. Coinciding with this visual loss, the extracellular matrix (ECM) is reorganized, which alters matrix metalloproteinase (MMP) activity levels. A potential pathological role of MMPs, MMP-9 in particular, involves an excitotoxicity-mediated physiological response. In the current study, we examine the MMP-9 and MMP-2 expression levels in the rhodopsin S334ter-line3 RP rat model and investigate the impact of treatment with SB-3CT, a specific MMP-9 and MMP-2 inhibitor, on rod cell survival was tested. Retinal MMP-9 and MMP-2 expression levels were quantified by immunoblot analysis from S334ter-line3 rats compared to controls. Gelatinolytic activities of MMP-9 and MMP-2 by zymography were examined. The geometry of rod death was further evaluated using Voronoi analysis. Our results revealed that MMP-9 was elevated while MMP-2 was relatively unchanged when S334ter-line 3 retinas were compared to controls. With SB-3CT treatment, we observed gelatinolytic activity of both MMPs was decreased and diminished clustering associated with rod death, in addition to a robust preservation of rod photoreceptors. These results demonstrate that up-regulation of MMP-9 in retinas of S334ter-line3 are associated with rod death. The application of SB-3CT dramatically interferes with mechanisms leading to apoptosis in an MMP-9-dependent manner. Future studies will determine the feasibility of using SB-3CT as a potential therapeutic strategy to slow progression of vision loss in genetic inherited forms of human RP.

## Introduction

Photoreceptor degenerative diseases affect millions of patients and diminish the ability of the retina to detect light and process visual signals. During retinal degeneration, retinal neurons are rewired while extracellular matrix (ECM) structural properties are changed. These changes alter matrix metalloproteinase (MMP) activity levels and influence cell-cell and cell-ECM interactions [1, 2]. More than 20 MMPs have been divided into collagenase (MMP-1, -8, and -13), gelatinases (MMP-2 and -9), stromelysins (MMP-3, -10, and -11), membrane-type MMPs (MT1- to MT6-MMP) and a heterogeneous MMPs (MMP-7, -12, -20, -26, and -28), based on their properties on the substrates [3]. Retinal degenerative diseases activate key members of the MMP family that contribute to complications [4–6]. For example, MMP-9 contributes to excitotoxicity-mediated pathogenesis [5, 7] and neurological disorders [8, 9]. Furthermore, in the retinal degeneration 1 (*rd1)* mouse retina, up-regulation of MMP-9 and MMP-2 has been documented [10]. In the past, efforts to reduce MMP-mediated retinal damage with broad-spectrum MMP inhibitors (e.g., GM6001) have produced encouraging results in animal models of retinal degeneration [5]. Inhibition of MMP-9 or well characterized downstream targets of the MMP-9 pathway prevents pathological remodeling of the inner limiting membrane and detachment-induced cell death of retinal ganglion cells (RGCs) [11, 12]. Furthermore, Chintala and colleagues (2002) reported that MMP-9 deficient mice are protected against retinal ganglion cell (RGC) death after optic nerve ligation.

Retinitis Pigmentosa (RP) begins with the death of rod photoreceptors and eventually leads to cone photoreceptor death [13]. Various treatment strategies in both RP patients and RP animal models include gene therapies [14–17], retinal pigment epithelium (RPE) [18], photoreceptor [19] and stem cell transplantation [20, 21]. In the initial stage of RP, external compounds, such as antioxidants or neurotrophic factors, protect photoreceptors because they are less invasive [22–25]. Basic fibroblast growth factor (bFGF) slows photoreceptor degeneration in Royal College of Surgeons (RCS) rat [26]. Ciliary neurotrophic factor (CNTF) delays photoreceptor degeneration in human retinal degeneration [25] and animal models such as *rd1* [27] and Q334ter mice [22]. However, the effectiveness of the drug treatment is also influenced by the health of retinal ECM [28]. With cell death in RP, there are a reduced overall number of integrin receptors at the ECM, which affects the oxygen levels, nutrients, and growth factors to the cells from the surrounding choroidal or retinal blood supplies [29].

In RP, rhodopsin S334ter-line3 (S334ter) rat retina, rods die in “clusters” [30–32], suggesting inductive cell death mechanisms consistent with animal models and human studies demonstrating that degenerating rods often lead to deaths of immediate neighbors [33–35]. Recently, we discovered Tissue Inhibitor of Metalloproteinase 1 (TIMP-1) restores the cone mosaic and protects cone outer segments at later stages of retinal degeneration in S334ter-line3 retina [32, 36, 37]. Although TIMP-1 influences MMP activity, it specifically binds to and inhibits MMP-9 activation [38]. The TIMPs consist of structurally and functionally distinct N- and C-terminal domains [39]. The N-terminal domain of a TIMP is for MMP inhibition [40]. In contrast, the C-terminal domain of TIMPs influences cell survival in an MMP-independent manner [41, 42]. There is evidence that TIMP-1 could enhance cell survival by directly suppressing apoptosis signaling pathways, in an MMP-independent manner [43, 44]. Thus, applying TIMP-1 cannot rule out the possibility that the C-terminal domain may involve in cell survival independently of MMP-9. In this study, we used SB-3CT, a highly selective inhibitor of MMP-2 and MMP-9 [2, 8, 45] to delay the death of rods in S334ter-line3 retina. The other synthetic MMP inhibitors (e.g., GM6001) [46–48], although highly successful in preclinical studies, lacked specificity and as a consequence failed due to adverse side effects [46–50]. The data in the present study demonstrate that SB-3CT treatment slows the death of rods and disrupts the cluster form of rod death in S334ter-line3 retina. In addition, MMP-9 is elevated and MMP-2 is relatively unchanged between control and S334ter-line3 retinas. These findings suggest that application of SB-3CT interferes with mechanisms leading to rod death in an MMP-9-dependent manner.

## Materials and Methods

### Animals

The third transgenic line of albino Sprague-Dawley (SD) rats homozygous for the truncated murine opsin gene (stop codon at Serine residue 334; S334ter-line-3) were originally provided by Matthew LaVail, Ph.D. (University of California, San Francisco, CA, USA). Homozygous S334ter-line-3 female rats were mated with Long Evans (LE) male rats to produce heterozygous offspring for the S334ter-line-3 transgene and referred to as the S334ter model. S334ter rats were euthanized at postnatal (P) days 15, 18, 30, 33, 37, 43, 45, and 60 (number of animals per group, 5 to 7 for each stage). For controls, age matched SD rats (Harlan, Indianapolis, IN, USA) were used. Controls were euthanized at P15, P33 and 37 (N = 5 for each stage). All rats were maintained on a daily 12/12-hour cycling light/dark cycle. The Veterinary Authority of University of Southern California and Use Committee reviewed and approved all procedures.

### Administration of SB-3CT

2-[(4-phenoxyphenyl)sulfonylmethyl]thiirane, SB-3CT, (EMD Millipore, Temecula, CA, USA) was prepared in phosphate buffered saline (PBS) with 0.05–0.1% dimethyl sulfoxide (DMSO) [51]. For preliminary testing of SB-3CT, 2 ml of several different final concentrations (10, 25 and 50 mg/ml) were injected into normal and S334ter rats at P30. Injection procedures were established and similar to our previously published studies [36, 37]. After preliminary testing, 25 ug/ml was used throughout this study. The developmental stage for the injection of SB-3CT was either P15, when there was peak rod death [52] and formation of dying rod clusters [31, 32], or P40, when cone rings were observed throughout the retina [36]. One eye was injected with SB-3CT and the other eye was injected with PBS with 0.05–0.1% DMSO for comparison for each animal. Induction of anesthesia was done by intraperitoneal (IP) injection of ketamine (20 mg/kg; KETASET, Fort Dodge, IA, USA) and xylazine (5 mg/kg, X-Ject SA; Butler, Dublin, OH, USA). Following injections, veterinary ophthalmic antibacterial ointment was applied to prevent drying of cornea and infection.

### Tissue preparation

Animals were anesthetized by IP injection of Euthasol (40mg/kg, Fort Worth, TX, USA) and the eyes were enucleated. Then, animals were euthanized by administration of an overdose of Euthasol. The cornea and lens were removed, and the eyecups were fixed in 4% paraformaldehyde for 90 minutes at 4°C. The eyecups were then transferred to 30% sucrose in 0.1 M phosphate buffer (PB) for 24 hours at 4°C. Following the procedure, the eyecups were frozen in liquid nitrogen, and stored at -70°C. The eyecups were embedded in Optimal Cutting Temperature embedding medium (Tissue-Tek, Elkhart, IN, USA) for cryostat section, then quickly frozen in liquid nitrogen and subsequently sectioned on a Leica cryostat at a thickness of 20μm. For whole mount preparation, the retinas were isolated from the eyecups and dissected as whole-mounts.

### Immunohistochemistry

The complete protocols for immunohistochemistry were previously published [31, 32, 36, 37]. Briefly, for retinal section immunohistochemical staining, 20 μm thick cryostat sections were incubated in 10% normal donkey serum (NDS) (Jackson ImmunoResearch Laboratories) for 1 hour at room temperature, and then incubated overnight with rabbit polyclonal antibody directed against glial fibrillary acidic protein (G9269, GFAP, Sigma-Aldrich Corp, dilution 1:500). Retinas were washed in PBS, and afterward incubated for 2 hours at room temperature in carboxymethylindocyanine (Cy3)-conjugated donkey anti-rabbit IgG (Jackson ImmunoResearch Laboratories, dilution 1:500). Next, the sections were washed with 0.1M PB, and cover slipped with Vectashield mounting medium (Vector Labs, Burlingame, CA, USA).

Similar procedures to the ones described above were used for whole-mount immunohistochemical staining;however, tissues were treated with 1% Triton X-100 in 0.1M PBS before 10% NDS (Jackson ImmunoResearch Laboratories) incubation to enhance antibody penetration. The whole-mounts were incubated with primary antibodies (rabbit polyclonal antibody directed against green opsin (M-opsin, dilution 1:2,000); mouse monoclonal antibody directed against rhodopsin (rho 1D4 [53], dilution 1:1,000)) in 0.5% Triton X-100 in 0.1M PBS for 48 hours at 4°C. After this incubation, the whole mounts were rinsed for 45 minutes with 0.1 M PBS. Afterward, they were incubated with corresponding secondary antibodies (carboxymethylindocyanine (Cy3) conjugated affinity-purified donkey anti-rabbit IgG or Alexa 488 conjugated donkey anti-mouse IgG (Molecular Probes, Eugene, OR, USA; dilution 1:300) for 24 hours at 4°C. The whole mounts were then washed again for 45 minutes with 0.1 M PB and cover slipped with Vectashield mounting medium.

A Zeiss LSM 710 (Zeiss, NY, USA) confocal microscope was used for the digital images, processed with the Zeiss LSM-PC software, and the brightness and contrast were adjusted by using Adobe Photoshop 7.0 (Adobe Systems, San Jose, CA, USA). All the adjustments were carried out equally across sections and whole mounts.

### TUNEL staining

Cell death was visualized using a In Situ Cell Detection kit (Boehringer Mannheim, Mannheim, Germany) according to manufactor’s recommendations. The sections and whole mounts were incubated with TUNEL reaction mixture (terminal deoxynucleotidyl transferase with nucleotide mixture in reaction buffer) for 90 minutes at 37°C. The sections or whole mounts were then washed for 30 minutes with 0.1 M PB and cover slipped with Vectashield mounting medium.

### Nuclei-positions map for TUNEL assay and M-cone distribution

The detailed instructions for evaluating the Nuclei-Position Map were previously published [36]. Confocal images of the retinas (n = 3–5 animals for each group) were taken at the focal level of the rod nuclei and M-cone nuclei, covering 1 x 1mm^2^ areas at the mid-peripheral region (3 mm away from optic disc) of the superior temporal retina. Each TUNEL stained nucleus or M-cone nucleus was marked with a white dot using the paint tool in Photoshop. Using these images, Voronoi domain and the coefficient of clustering (CC) were also analyzed.

### Voronoi analysis

The detailed instructions for the Voronoi analysis was previously published [36, 54]. Briefly, the Voronoi domain of each dying rod or M-cone was generated, and then the areas of each polygon were calculated and plotted on a histogram for the Voronoi analysis. The coefficient of clustering (CC) was also determined as previously described [36, 54].

### Immunoblot analysis

The detailed instructions for the immunoblot analysis were published in a recent study [55]. Briefly, 45 μg of protein per retina were electrophoresed on the 10% SDS-PAGE, transferred to nitrocellulose membranes (LI-COR Biotechnology, Lincoln, NE). After 1 hour of protein blocking with Odyssey blocking buffer (LI-COR Biotechnology), membranes were incubated overnight sequentially with primary antibodies for anti β-actin (A5316 Sigma, 1:4000) and either anti-MMP-9 (MAB3309 Millipore, 1:500), or anti-MMP-2 (sc-8835 Santa Cruz, 1:100). Appropriate secondary antibodies with a fluorophore (680 nm or 800 nm) were used for detection under the infrared detection system (GENESys, Syngene, Frederick, MD). For all optical density analysis, we used NIH Image J software version 1.50i to quantify the intensity of each band. Relative amounts of the MMP-9 and MMP-2 were calculated by dividing the intensity of the MMP-9 or MMP-2 band by the intensity of the β -actin band. The average of the normal and saline-treated S334ter was set as 100%.

### Gelatin zymography

Samples were prepared as described above for immunoblot analysis. 45 μg of retinal protein extracts were mixed with zymogram loading buffer (Novex Tris Glycine SDS Sample buffer, Novex Life Technologies, Carlsbad, CA) without boiling and applied to 10% NOVEX Pre-Cast SDS polyacrylamide gel (Novex Life Technologies) in the presence of 0.1% gelatin under non-reducing conditions for electrophoresis. Positive controls for MMP-9 and MMP-2 included 1.5 ng of recombinant mouse MMP-9 (AnaSpec, Fremont, CA) and 6.6 ng of recombinant mouse/rat MMP-2 (R&D Systems, Minneapoilis, MN), respectively. After electrophoresis, the gels were washed with deionized water, and then each was incubated in zymogram renaturing buffer (Novex Life Technologies) for 30 minutes at room temperature and then developed in zymogram developing buffer (Novex Life Technologies) for 16 hours at 37°C to allow proteolysis of the substrates in the gels. After staining with SimpleBlue^™^ Safestain (Novex Life Technologies) for 1 hour, gels were de-stained in deionized water for 1 hour and imaged. Images were scanned using HP Photosmart 7520 and processed using Photoshop CC (Adobe, San Jose, CA) software.

### Statistical analysis

All the statistics were presented as mean + standard error of the mean (SEM). Student’s t-test, two-way ANOVA and Fisher's least significant difference procedure (LSD test) were used to examine the differences among the groups. To perform the test and generate graphs, GraphPad Prism 6 (La Jolla, CA, USA) was used. The difference between the means of separate experimental conditions was considered statistically significant at p< 0.05.

## Results

### Expression of MMP-9 and MMP- 2 in the S334ter retina

**Fig 1:**
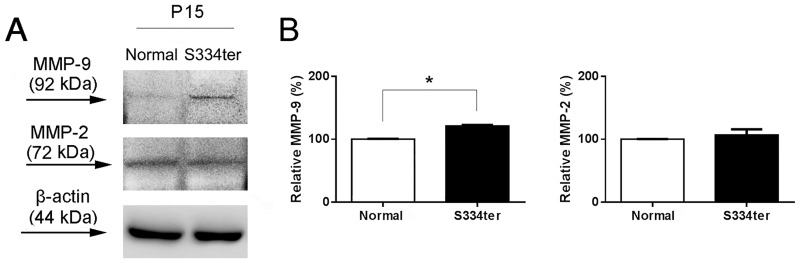
Expression of MMP-9 and MMP- 2 in the S334ter retina. Immunoblot analysis of MMP-9 and MMP-2 immunoreactive protein levels in the P15 normal and P15 S334ter rat retinas. Immunoblots were processed with specific primary antibodies stained for MMP-9 and MMP-2, demonstrating a single band at 92 kDa (arrow) and 72 kDa (arrow), respectively (A). Immunoblot analysis reveals up-regulation of MMP-9 in the S334ter retina. In contrast, MMP-2 was not affected. Immunodetection using a primary antibody for beta-actin was used to evaluate equal protein loading as a control (44kDa). Densitometry analysis of immunoblots as shown in B. Data represent mean + SEM; *p < 0.01.

Increased MMP-9 or MMP-2 expression is associated with retinal degenerative diseases [3, 5, 10, 56]. To determine whether rod cell death in S334ter is mediated by MMPs in our rat model, we first investigated the expression levels of MMP-9 and MMP-2 in retinal extracts by immunoblot analysis using specific antibodies against MMP-9 and MMP-2. The MMP-9 (92 kDa) and the MMP-2 (72 kDa) immunoreactive bands of (Fig 1A) were identified in retinal extracts of P15 normal, which is consistent with a previous study showing single bands in normal mouse retina [5]. Our data showed that MMP-9 is elevated (Fig 1A) while MMP-2 is relatively unchanged between normal and S334ter retinas. Densitometry of these MMP-9- and MMP-2-immunoreactive proteins was performed (Fig 1B). The MMP-9 protein levels were ~20% higher in P15 S334ter retinas than in P15 normal retinas (unpaired, two-sided t-test, n = 3 normal and n = 5 S334ter retinas; P < 0.01). In contrast, there were no significant differences in the expression levels of MMP-2 between normal and S334ter retinas (Fig 1B). Beta-actin expression was probed with an antibody as a loading control. Our data indicated that only MMP-9 expression was elevated at this early stage of degeneration in the S334ter retina.

### Absence of glial activation and cell death with SB-3CT treatment

**Fig 2:**
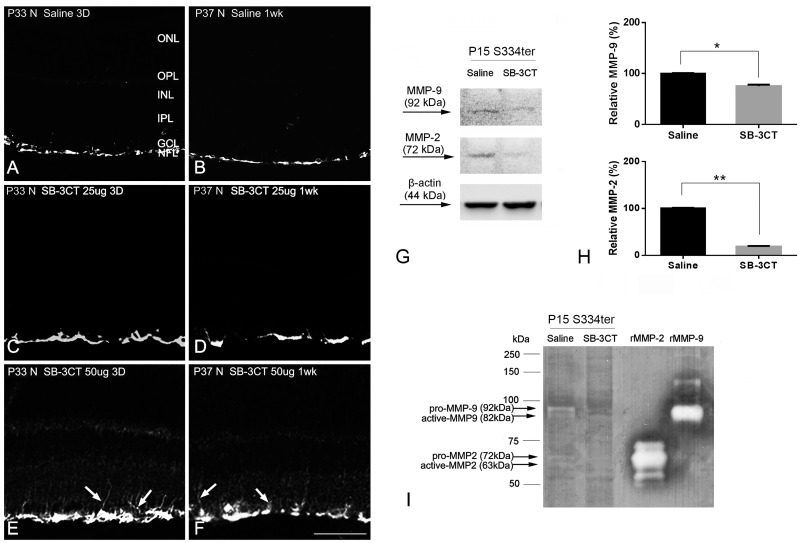
Absence of glial activation and cell death with SB-3CT treatment. Confocal micrographs taken from frozen cryostat sections of normal retinas processed for GFAP immunoreactivity shown for saline-treated groups (A, B) and SB-3CT treated groups (C-F) after 3 days and after 1 week post-injection. The saline and SB-3CT were injected at P30. The saline (A, B) and 25 ug/ml SB-3CT (C, D), caused no significant up-regulation of GFAP expression. 50 ug/ml SB-3CT showed modest up-regulation of GFAP expression (E, F, arrows). Retinal proteins were extracted 12 hours after intravitreal injection of saline or 25 ug/ml SB-3CT (G-I). In the immunoblot analysis, the level of MMP-9 (92kDa) and MMP-2 (72kDa) were significantly decreased in SB-3CT treated retina after 12 hours post-injection (G, H). In the gelatin zymography, SB-3CT attenuated the level of pro-MMP-9 (92 kDa), active MMP-9 (82 kDa), pro-MMP-2 (72 kDa) and active MMP-2 (63 kDa) in S334ter (I). Recombinant mouse MMP-9 and recombinant mouse/rat MMP-2 were applied to the gel and transferred to the membrane as positive controls. P, postnatal; D, days; wk, week; N, normal; ONL, outer nuclear layer; OPL, outer plexiform layer; INL, inner nuclear layer; IPL, inner plexiform layer; GCL, ganglion cell layer; NFL, nerve fiber layer. Scale bar = 50 um. Data are presented as mean + SEM. The symbol * and ** indicates p<0.05 and p<0.01, respectively.

SB-3CT is a specific inhibitor of MMP-2 and MMP-9 [2, 8, 45]. First, the safety of SB-3CT in concentrations used for intravitreal injections (10, 25 and 50 ug/ml) was tested at P30. To check if SB-3CT was toxic to retinal cells, normal retinas from the saline- and the SB-3CT-treated groups were immunohistologically stained with GFAP, a marker for glial activation [57], after 3 days and after 1 week post-injection. The controls showed GFAP expression in the nerve fiber layer (NFL, Fig 2A and 2B), and there was no significant up-regulation of GFAP expression at 3 days or 1 week in 10 ug/ml (data not shown) and 25 ug/ml groups (Fig 2C and 2D). 50 ug/ml SB-3CT treated groups showed a moderate up-regulation of GFAP expression (Fig 2E and 2F, arrows). There were no TUNEL-positive cells in any group especially 25μg/mL injected group (S2 Fig). Thus, 25 ug/ml SB-3CT was used for all following additional studies.

To determine if administration of SB-3CT treatment (25 ug/ml) inhibited MMP-9 and MMP-2 expression and activity in S334ter retina, we used immunoblot analysis (Fig 2G and 2H) and zymography (Fig 2I), respectively. SB-3CT was injected at P15 and retinal extracts were collected 12 hours after injection because MMP-9 activity showed maximum changes between 6 hours and 24 hours after the injection (5, 9). The 12 hours-post injection of SB-3CT-treated S334ter retinal lysates showed that the expression levels of MMP-9 (92kDa) and MMP-2 (72kDa) significantly decreased (Fig 2G and 2H). Gelatin zymography on the saline-treated retinal lysates showed both pro-MMP-9 (92kDa) and active-MMP-9 (82kDa) bands. In addition, both pro-MMP-2 (72kDa) and active-MMP-2 (63kDa) bands were present in retinal lysates of saline-treated S334ter retina. Gelatinolytic activity of pro-MMP-9, active-MMP-9, pro-MMP-2, and active-MMP-2 were also significantly decreased in SB-3CT-treatd P15 S334ter retina (Fig 2I). Recombinant mouse MMP-9 and recombinant mouse/rat MMP-2 were used as loading controls. We also performed gelatin zymography on retinal lysates of P15 (12hours after the injection), P30, P45, and P60 saline- and SB-3CT treated S334ter retinas. The SB-3CT was injected at P15 and retinal lysates were collected at P15, P30, P45, and P60 (S3 Fig). In later stages of S334ter retinas, we observed no detectable activity of MMP-9 and MMP-2 (S3 Fig). In summary, SB-3CT dramatically inhibits MMP-9 and MMP-2 activities in early stage of S334ter retina (i.e. P15). In addition, the activity of MMP-9 and MMP-2 diminished after peak rod death at P15 [52].

### SB-3CT treatment delays rod death in S334ter retina

**Fig 3:**
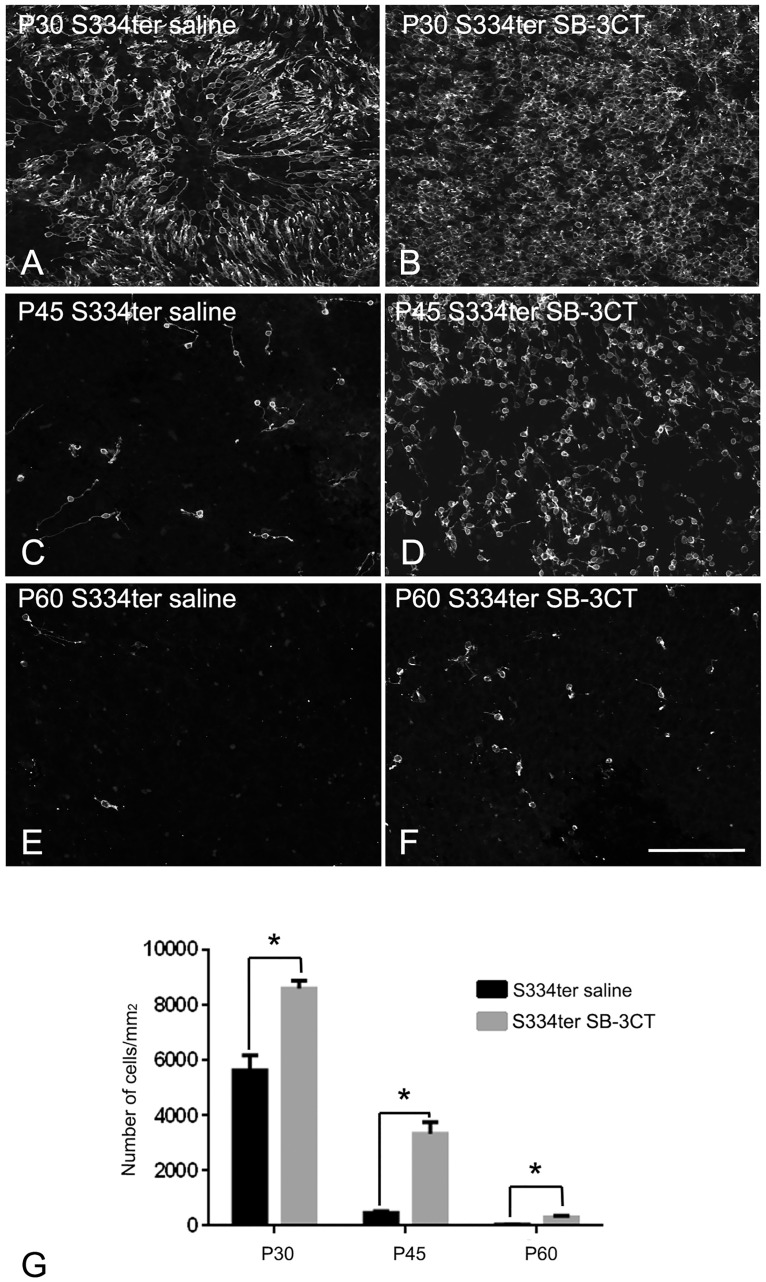
SB-3CT treatment delays rod death in S334ter retina. Confocal micrographs of whole-mounts labeled with rhodopsin in saline-treated S334ter retina (A, C and E) and SB-3CT treated S334ter retina (B, D and F) at P30, P45 and P60 in the central (1mm away from optic disc) retina. Rod number per 1mm^2^ in SB-3CT treated S334ter retina was significantly higher than age matched saline-treated S334ter retina at P30, P45, and P60 (G). Data are presented as mean + SEM. The symbol * indicates p<0.05. P, postnatal, Scale bar = 100 um.

To determine if inhibition of up-regulated MMP-9 via SB-3CT (Figs 1 and 2) can affect cell survival, we injected either SB-3CT (25 ug/ml) or saline (for control) to P15 S334ter rats. After a single injection at P15, animals were sacrificed at P30, P45 and P60. For quantitative analysis, rhodopsin-immunoreactive stained cells were counted at each time point. Fig 3A–3F shows an example of a whole-mount processed for rhodopsin immunoreactivity at P30 (Fig 3A and 3B), P45 (Fig 3C and 3D), and P60 (Fig 3E and 3F) taken at the central part of saline-treated (Fig 3A, 3C and 3E) and SB-3CT treated (Fig 3B, 3D and 3F) retinas. Consistent with our previous work, we observed holes in the rod mosaic in P30 saline-treated S334ter retina (Fig 3A [32]). In later stages of the degeneration, fewer and scattered cells were observed (Fig 3C and 3E). In contrast, rods in P30 SB-3CT-treated S334ter retina were more homogeneously distributed (Fig 3B). In P30, P45 and P60 retinas, more rods were detected in SB-3CT treated groups compared to the age matched saline-treated groups (Fig 3B, 3D and 3F). The summary graph illustrates the mean rod density (Fig 3G) measured from the 1x1 mm^2^ sampling areas (for details, see methods) of saline-treated and SB-3CT-treated S334ter retinas. The mean density of cells in saline–treated S334ter retinas at P30, P45, and P60 were 5,640±551, 469±63 and 33±5 cells/mm^2^, respectively. The density of cells changed with SB-3CT–treated S334ter groups. The density from the SB-3CT–treated S334ter retinas showed higher numbers of 8,611±296, 3,348±417, and 304±61 cells/mm^2^ at P30, P45, and P60, respectively (Fig 3G). The two-way ANOVA analysis showed significant differences between the mean of different groups and the different postnatal days (Fig 3G; p < 0.001). These data clearly demonstrate that SB-3CT treatment substantially enhances survival by delaying rod death in S334ter retina.

### Disruption of cluster-form of rod deaths with SB-3CT in S334ter retinas

**Fig 4:**
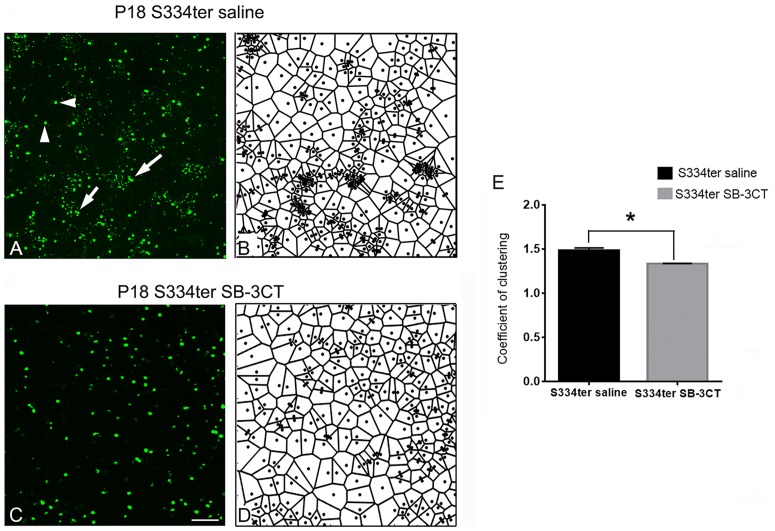
Disruption of cluster-form of rod deaths with SB-3CT in S334ter retinas. Confocal micrographs of whole-mounts labeled with TUNEL staining in saline-treated S334ter (A) and SB-3CT treated S334ter (C) retina at P18. Corresponding Voronoi domains are shown for each Fig (B, D). While S334ter retina showed clusters of cell death (A, arrows, B), SB-3CT treated S334ter retina showed a random distribution of rod cell death (C, D). Coefficient of clustering (CC) was significantly different within the two groups (E). Data are presented as mean + SEM. The symbol * indicates p<0.05. P, postnatal, Scale bar = 50 um.

In rhodopsin S334ter-line3 RP retina, rods die in clusters and create holes in the rod mosaic in the early stage of retinal degeneration and the resulting pattern triggers the formation of cone rings [31, 32]. In Fig 3A, we observed a more homogenous distribution of rods in SB-3CT-treated retina. Thus, we investigated if SB-3CT disrupts cluster-form of rod death to prevent holes in the rod mosaic. The presence of the cluster-form of rod death was described around P18 [32]. Thus, we injected SB-3CT at P15 and observed the effects of SB-3CT after 3 days of saline- or SB-3CT post-injection. Saline-treated and SB-3CT-treated retinas at P18 were stained with TUNEL (Fig 4A and 4C). As expected in the saline-treated retina, cluster-form of cell death appeared (Fig 4A). [31, 32]. In SB-3CT-treated retinas, TUNEL positive cells were randomly distributed (Fig 4C). Furthermore, when we used Voronoi analysis to determine the geometry of rod death, clusters of rod death are distinguishable from the random distribution of rod death in the saline-treated P18 retina (Fig 4A, arrowheads). Illustrating this morphological distribution with the Voronoi diagram, we observed that most of smaller domains were closer to other small domains, while most of larger domains were surrounded by larger domains (Fig 4B). After 3 days of SB-3CT post-injection, we no longer observed clusters of rod death. TUNEL staining showed a homogenous distribution of rod death (Fig 4C). Consistently, the Voronoi diagram showed only a mixture of large and small domains (Fig 4D). We quantified the correlation between the sizes of neighboring domains by calculating the coefficient of clustering (CC) [36]. If the small domains or large domains are aggregated together CC would be greater than 1. If instead, Voronoi domain showed random distribution, the CC would be near 1. The CC was high in saline-treated (1.48 ± 0.03) and became significantly lower with SB-3CT injection (1.32 ± 0.006) (Fig 3E, p = 0.006). Therefore, the pattern of dying rods became less clustered upon SB-3CT injection.

**Fig 5:**
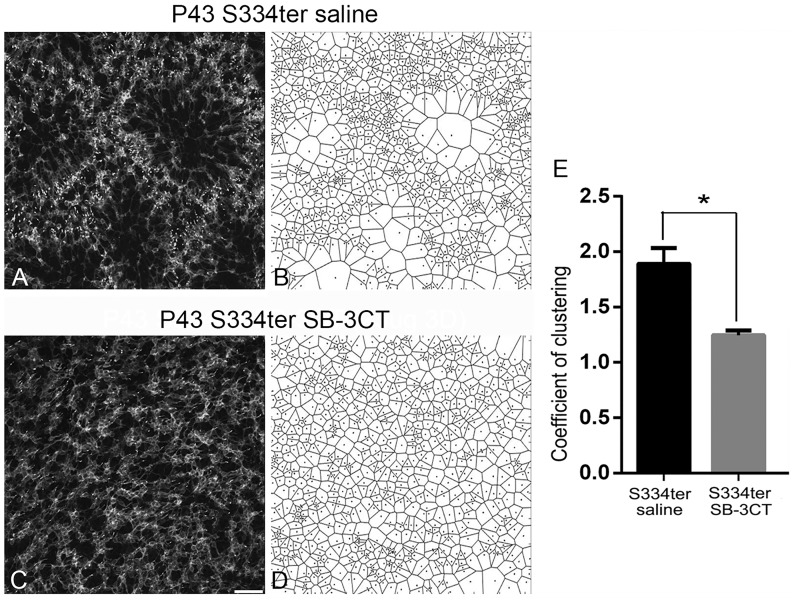
Disruption of cone rings with SB-3CT in S334ter retinas. Confocal micrographs of whole-mounts for M-opsin immunohistochemical staining of saline-treated S334ter (A), and SB-3CT treated S334ter retina (C). Saline and SB-3CT (25 ug/ml) are injected at P40. Cones in SB-3CT treated S334ter retinas (C) have rearranged into a homogeneous pattern 3 days after injection, while cones in S334ter retinas showed a ring-like pattern in the superior temporal region at the same age (A). Corresponding Voronoi domains are shown for each Fig (B, D). While S334ter retina showed ring-like pattern (A, B), SB-3CT treated S334ter retina showed a random distribution of cones (C, D). Coefficient of clustering was significantly different within the two groups (E). Data are presented as mean + SEM. The symbol * indicates p<0.05. P, postnatal, Scale bar = 50 um

To examine if application of SB-3CT can induce a homogeneous cone mosaic, we injected SB-3CT at P40 and analyzed the cone mosaic 3 days post-injection (i.e. P43). By P40, cone rings were observed throughout the entire retina [36]. The positive staining of M-opsin cones were immunolabeled in the S334ter whole-mount retinas of the saline-treated (Fig 5A) and the SB-3CT-treated (Fig 5C) groups. Voronoi analysis on S334ter retinas of saline-treated (Fig 5B) and SB-3CT-treated (Fig 5D) was performed to quantify changes in the cone mosaic (i.e. disappearance of cone rings). In the S334ter saline-treated retinas, most Voronoi domains were small, as cell bodies of M-cone are distributed around the rim of the rings. A few large Voronoi domains were observed in the center of the rings (Fig 5B). In contrast, in SB-3CT–treated retinas, Voronoi domains with extremely large sizes are reduced, and cones became more homogeneous after 3 days of post-injection (Fig 5D). Our results showed significant differences in CC between saline treated (1.89 ± 0.08) and SB-3CT-treated (1.24 ± 0.02) S334ter retinas (Fig 5E, p = 0.003). In summary, SB-3CT induced mosaics of M-cones in S334ter retinas to maintain and potentially restore homogeneity.

## Discussion

### Up-regulation of MMP-9 protein expression levels in S334ter retina

Inherited human retinal degenerative diseases are induced by mutations in over 190 genes [58, 59], including some genes in ECM-specific proteins [1]. In past studies, it was reported that changes in the properties of the ECM affect the function of a different types of cells and modulate the synthesis and release of MMPs/TIMPs [60, 61].

In this study, we demonstrated that MMP-9 is linked to and contributes to rod death in the RP associated with the S334ter retina (Figs 1, 2 and 3). The MMP-9 immunoreactive protein levels were ~20% higher in P15 S334ter retinas than in P15 normal retinas. P15 is the stage that has the highest number of apoptotic cells in the outer nuclear layer (ONL) in S334ter retina [52]. In contrast, there were no significant differences in the levels of expression of MMP-2 between normal and S334ter retinas (Fig 1). These data are different from a previous study showing up-regulation of both MMP-9 and MMP-2 in *rd1* mouse retina model [10]. The difference is unclear; however, in *rd1* retina they observed that MMP-2 was significantly higher at P2 than that in later stage (e.g., P14). Furthermore, MMP-2 activity/expression was reported to remain unchanged in other retinal degeneration models such as excitotoxic injury, ischemia-reperfusion (IR) injury or optic nerve transection [11, 62, 63]. Hence, some discrepancies between the *rd1* and our study may be related to different animal disease models or the rapid onset of retinal degeneration at an earlier postnatal time.

Although the precise mechanisms that lead to up-regulation of MMP-9 in S334ter are not clear at this time, previous studies have suggested that the reorganization of the ECM during the degenerative process may influence MMP activity because the levels of MMPs are modulated by cell-cell and cell-ECM interactions [2]. Alternatively, up-regulation of MMP-9 in S334ter may be due to glutamate-induced toxicity in RP [64]. Glutamate is known to be involved in the activation of MMP-9 [65]. Delyfer and coworkers (2005) reported that photoreceptor degeneration was associated with excessive free glutamate levels and with an up-regulation of glutamate turnover in *rd1* mouse retinas. Thus, we hypothesize that up-regulation of MMP-9 is resulted by glutamate toxicity in S334ter. Further studies are needed in order to test and verify this hypothesis.

### Delaying rod death with SB-3CT

We first confirmed that SB-3CT treatment inhibited MMP-9 (and MMP-2) expression and activities by performing immunoblot analysis and gelatin zymography (Fig 2), respectively. SB-3CT attenuated the level of pro- and active- forms of both MMP-9 and MMP-2 in S334ter after 12 hour post-injection. The results presented in this study are also consistent with other recent work in the central nervous system [66–68]. In our study, the inactive-form of MMP-2 and MMP-9 was not detected on the immunoblot analysis, whereas the level of their expression was detected by zymography under the same experimental conditions in retinal extracts. The apparent discrepancy between the zymography and immunoblot analysis could be explained by the sensitivity and detection of the methods. The sensitivity of detecting MMPs with immunoblot analysis is lower than with zymography [69–71]. Thus, relative absence of inactive-forms of MMP-2 and MMP-9 on immunoblot analysis does not rule out the presence of inactive-forms.

Our results showed that treatment with SB-3CT delays rod death in S334ter retinas (Fig 3). At P30, many rods have died [32, 52] while the number of cones remains similar in normal and S334ter retinas even at an older age (P180) [30]. The activity of MMP-9 in a later stage of S334ter treatment (i.e. P30, P45, P60), retinas did not show significant induction compared to that of P15 S334ter retinas (S3 Fig). The results suggest that up-regulation of MMP-9 may be associated with a retinal stage of active cell death. Our results, clearly demonstrated that inhibition of up-regulated MMP-9 in P15 (peak cell death) was significant enough to cause effects on the number of rods at the later stage of S334ter retinas (Figs 2, 3 and S3). Thus, exogenous application of SB-3CT at peak stage of cell death (P15) may interfere with the mechanisms of rod death in an MMP-9-dependent manner. The exogenous application of SB-3CT in S334ter may counterbalance the increased MMP-9 to slow the rod death. Increased expression levels of MMP-9 are associated in several pathological conditions such as neuronal cell death in glaucoma [3, 11, 72], cerebral ischemia [8], diabetic retinopathy [4], injured peripheral nerves [73], and retinal degeneration [5, 11] including RP [10]. Up-regulated MMP-9 in these various diseases may lead to significant cell death by degrading ECM, thereby interfering with integrin-mediated survival signaling [5, 7, 8, 72, 74, 75]. Inhibition of MMP-9 is also known to prevent cell death [5, 11, 12]. Therefore, it is likely that SB-3CT modulates both apoptotic and non-apoptotic pathways [76] by interfering with MMP-9 activity in ECM.

Alternatively, SB-3CT treatment may slow rod death progression by disrupting the cluster-form of rod death in the S334ter retina (Fig 4) [31, 32]. These dramatic changes in rod cell death distribution may also due to an imbalance in the levels of MMPs and TIMPs in RP that potentially modify the intercellular and cell-ECM interactions [72]. Thus, in our study, SB-3CT treatment may have modified further intercellular and surviving rod-ECM interactions resulting in a rearrangement of rod patterns. The cluster patterns of cell death suggest an inductive mechanism of cell death [33–35]. For example, if the cluster-form of rod death was caused by transmission of toxic substances via gap junctions connecting the adjacent cells, disrupting the cluster-form of cell death with SB-3CT will prevent the detrimental effects on neighboring cells [34, 77]. Thus, SB-3CT may be a potential therapeutic agent to slow progression of human forms of inherited RP if we can control the spreading effect with SB-3CT.

### Rearrangement of cones with SB-3CT

What are the possible mechanisms that may contribute to the underlying modulation of cell rearrangement with SB-3CT? Our hypothesis is that SB-3CT actively and efficiently inhibits MMP-9, which then induces cell rearrangement. Our results demonstrate that SB-3CT dramatically changes the cone mosaic in S334ter retinas (Fig 5). We predict that further remodeling of cone distribution modified the ECM proteins (i.e., laminin) essential for cell movement [78, 79]. Laminin in the ECM is an MMP-9 degradation target [80]. In support of this, application of SB-3CT blocks this function of MMP-9, thereby supporting cell movement [72, 80, 81].

## Conclusion

We have clearly demonstrated that SB-3CT treatment disrupts the clustering pattern associated with rod death and produces robust preservation of rod photoreceptors. Our study provides novel insights into how SB-3CT works in the animal model of RP. Our findings have potential therapeutic implications and may provide a future treatment with SB-3CT, which could simultaneously promote photoreceptor survival and maintain homogeneous distribution of cone photoreceptors.

## Supporting Information

S1 Fig Example of P43 SB-3CT-treated S334ter nuclei position map. Legend: Nuclei positions map was constructed by marking the location of cell bodies using white dots. Applying white dot allowed identification of the position of each M-opsin positive cell in the retinal area. Also, using these images, Voronoi domain and the coefficient of clustering was measured.
(DOCX)

S2 Fig TUNEL staining in SB-3CT-treated normal retina. Legend: TUNEL staining in 25ug/ml SB-3CT treated groups after 3 days (A) and after 1 week (B) post-injection. There were no TUNEL positive cells in either time-point. P, postnatal; D, days; wk, week; N, normal; ONL, outer nuclear layer; OPL, outer plexiform layer; INL, inner nuclear layer; IPL, inner plexiform layer; GCL, ganglion cell layer. Scale bar = 50 um.
(DOCX)

S3 Fig Reduction of MMP-9 and MMP-2 activities in a later stage of S334ter retina. In the gelatin zymography, SB-3CT attenuated the level of pro-MMP-9 (92 kDa), active MMP-9 (82 kDa), pro-MMP-2 (72 kDa) and active MMP-2 (63 kDa) in P15 S334ter. In P30, P45, and P60, no activity of MMP-9 and MMP-2 was observed in both saline (-) and SB-3CT (+) treated retinas. Recombinant mouse MMP-9 and recombinant mouse/rat MMP-2 were applied to the gel and transferred to the membrane as positive controls.
(DOCX)

S1 Table Quantification of MMP-9 and MMP-2 expression in normal vs S334ter retina by immunoblot analysis. Legend: Immunoblot analysis shows up regulation of MMP-9 in the S334ter rat retina, compared to normal retina. Beta actin was used as loading control to obtain relative MMP-9 and MMP-2 expression.
(DOCX)

S2 Table The mean density of rods in saline–treated and SB-3CT-treated S334ter retinas. Legend: The mean rod density was measured from the 1x1 mm^2^ sampling areas (for details, see methods) of saline-treated S334ter and SB-3CT-treated S334ter retinas.
(DOCX)

S3 Table The coefficient of clustering of TUNEL positive cells in P18 saline–treated and P18 SB-3CT-treated S334ter retinas. Legend: The mean coefficient of clustering was measured in all groups (Fig 4).
(DOCX)

S4 Table The coefficient of clustering of M-opsin cones in P43 saline–treated and SB-3CT-treated S334ter retinas. Legend: The mean coefficient of clustering was measured in all groups (Fig 5).
(DOCX)

S5 Table Cone coordinates of P43 SB-3CT S334ter retinas. Legend: The x and y are the coordinates of cones extracted from white-dot images. All the cone mosaic analyses are based on the coordinates.
(XLSX)
