# Supplementary figures and images for: Inhibition of Matrix Metalloproteinase 9 Enhances Rod Survival in the S334ter-line3 Retinitis Pigmentosa Model
Source: PLoS One. 2016 Nov 28;11(11):e0167102. doi: 10.1371/journal.pone.0167102 (PMC5125676; doi:10.1371/journal.pone.0167102)

S1 Figure


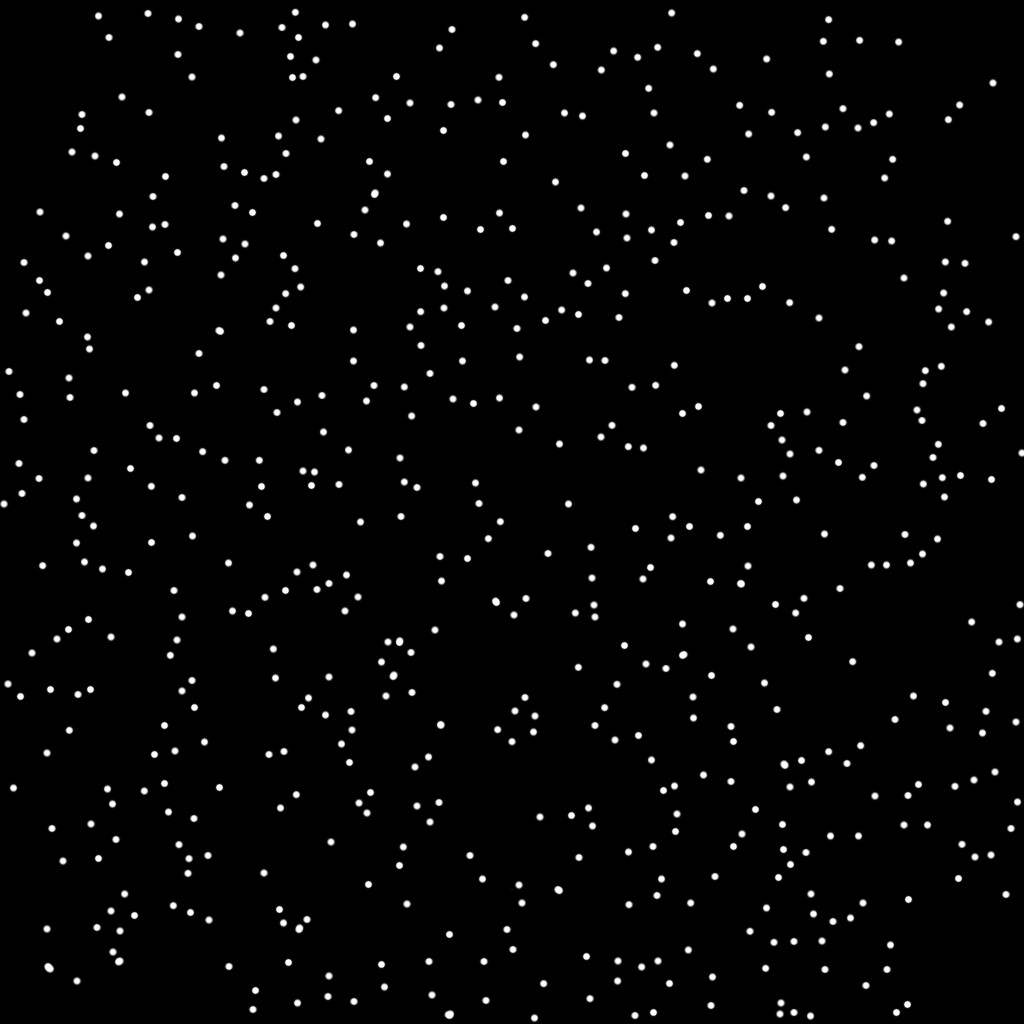

Supplement: S1 Fig — Legend: Nuclei positions map was constructed by marking the location of cell bodies using white dots. Applying white dot allowed identification of the position of each M-opsin positive cell in the retinal area. Also, using these images, Voronoi domain and the coefficient of clustering was measured. (DOCX) [file pone.0167102.s001.docx]

S2 Figure


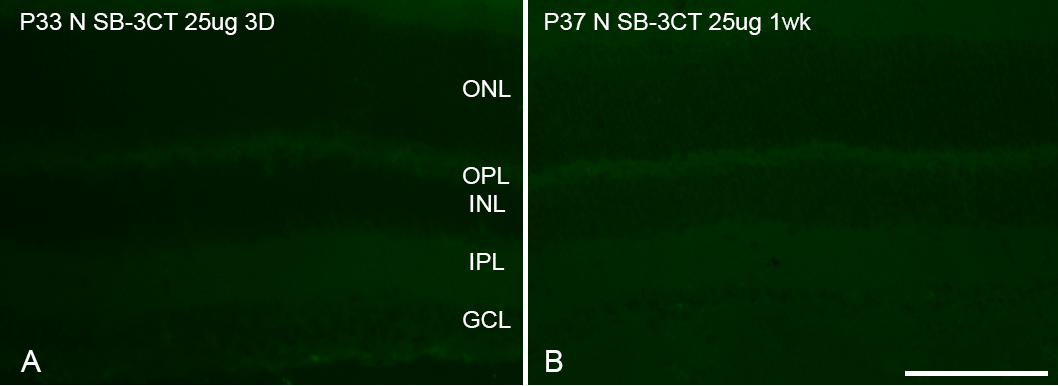

Supplement: S2 Fig — Legend: TUNEL staining in 25ug/ml SB-3CT treated groups after 3 days (A) and after 1 week (B) post-injection. There were no TUNEL positive cells in either time-point. P, postnatal; D, days; wk, week; N, normal; ONL, outer nuclear layer; OPL, outer plexiform layer; INL, inner nuclear layer; IPL, inner plexiform layer; GCL, ganglion cell layer. Scale bar = 50 um. (DOCX) [file pone.0167102.s002.docx]

S3 Figure


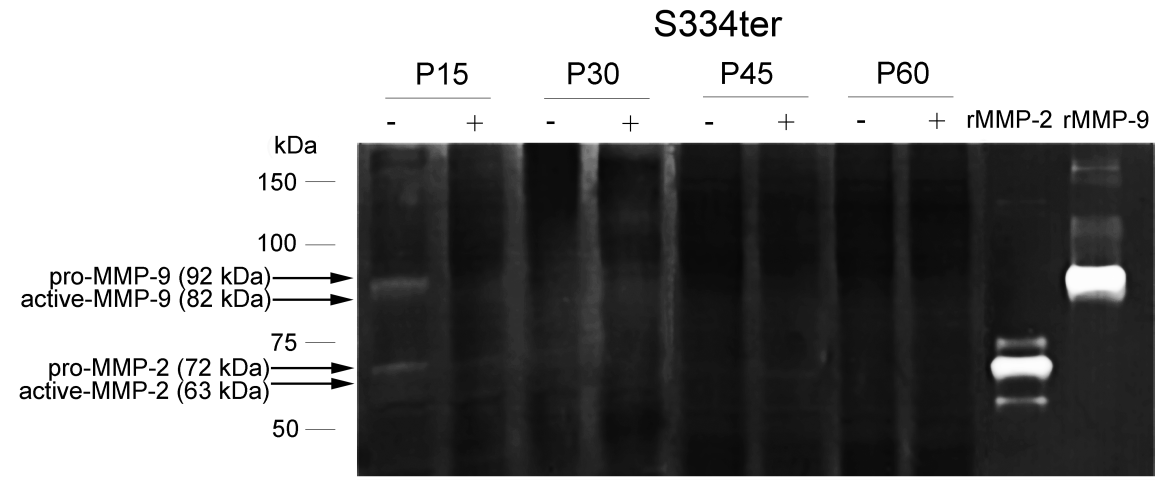

Supplement: S3 Fig — In the gelatin zymography, SB-3CT attenuated the level of pro-MMP-9 (92 kDa), active MMP-9 (82 kDa), pro-MMP-2 (72 kDa) and active MMP-2 (63 kDa) in P15 S334ter. In P30, P45, and P60, no activity of MMP-9 and MMP-2 was observed in both saline (-) and SB-3CT (+) treated retinas. Recombinant mouse MMP-9 and recombinant mouse/rat MMP-2 were applied to the gel and transferred to the membrane as positive controls. (DOCX) [file pone.0167102.s003.docx]
