# Supplementary material for: Inhibition of Matrix Metalloproteinase 9 Enhances Rod Survival in the S334ter-line3 Retinitis Pigmentosa Model
Source: PLoS One. 2016 Nov 28;11(11):e0167102. doi: 10.1371/journal.pone.0167102 (PMC5125676; doi:10.1371/journal.pone.0167102)
Supplement: S1 Table — Legend: Immunoblot analysis shows up regulation of MMP-9 in the S334ter rat retina, compared to normal retina. Beta actin was used as loading control to obtain relative MMP-9 and MMP-2 expression. (DOCX) [file pone.0167102.s004.docx]

| S1 Table  Relative MMP-9 |  |  |  |
| --- | --- | --- | --- |
|  | Sample 1 | Sample 2 | Sample 3 |
| Normal | 100.6911 | 100.0962 | 100.7102 |
| S334ter | 122.4151 | 120.2254 | 120.4589 |

| Relative MMP-2 |  |  |  |
| --- | --- | --- | --- |
|  | Sample 1 | Sample 2 | Sample 3 |
| Normal | 99.94103 | 100.54210 | 100.60020 |
| S334ter | 100.64140 | 113.25860 | 108.83110 |
