# Supplementary material for: Inhibition of Matrix Metalloproteinase 9 Enhances Rod Survival in the S334ter-line3 Retinitis Pigmentosa Model
Source: PLoS One. 2016 Nov 28;11(11):e0167102. doi: 10.1371/journal.pone.0167102 (PMC5125676; doi:10.1371/journal.pone.0167102)
Supplement: S2 Table — Legend: The mean rod density was measured from the 1x1 mm2 sampling areas (for details, see methods) of saline-treated S334ter and SB-3CT-treated S334ter retinas. (DOCX) [file pone.0167102.s005.docx]

| S2 Table |  |  |  |
| --- | --- | --- | --- |
|  | Sample 1 | Sample 2 | Sample 3 |
|  | rod density | rod density | rod density |
| P30 S334ter saline | 4797 | 6677 | 5446 |
| P30 S334ter SB-3CT | 8603 | 8102 | 9127 |
| P45 S334ter saline | 561 | 498 | 349 |
| P45 S334ter SB-3CT | 3486 | 3992 | 2568 |
| P60 S334ter saline | 24 | 38 | 38 |
| P60 S334ter SB-3CT | 249 | 425 | 238 |
