# Supplementary material for: Inhibition of Matrix Metalloproteinase 9 Enhances Rod Survival in the S334ter-line3 Retinitis Pigmentosa Model
Source: PLoS One. 2016 Nov 28;11(11):e0167102. doi: 10.1371/journal.pone.0167102 (PMC5125676; doi:10.1371/journal.pone.0167102)
Supplement: S3 Table — Legend: The mean coefficient of clustering was measured in all groups (Fig 4). (DOCX) [file pone.0167102.s006.docx]

| S3 Table |  |  |  |
| --- | --- | --- | --- |
|  | Sample 1 | Sample 2 | Sample 3 |
|  | coefficient of clustering | coefficient of clustering | coefficient of clustering |
| P18 S334ter saline | 1.501873 | 1.523507 | 1.425088 |
| P18 S334ter SB-3CT | 1.314671 | 1.314074 | 1.333778 |
