# Supplementary material for: Inhibition of Matrix Metalloproteinase 9 Enhances Rod Survival in the S334ter-line3 Retinitis Pigmentosa Model
Source: PLoS One. 2016 Nov 28;11(11):e0167102. doi: 10.1371/journal.pone.0167102 (PMC5125676; doi:10.1371/journal.pone.0167102)
Supplement: S4 Table — Legend: The mean coefficient of clustering was measured in all groups (Fig 5). (DOCX) [file pone.0167102.s007.docx]

S4 Table

|  | Sample 1 | Sample 2 | Sample 3 | Sample 4 |
| --- | --- | --- | --- | --- |
|  | coefficient of clustering | coefficient of clustering | coefficient of clustering | coefficient of clustering |
| P43 S334ter saline | 1.725075 | 1.984047 | 1.964449 |  |
| P43 S334ter SB-3CT | 1.30489 | 1.24731 | 1.22188 | 1.20244 |
